# Supplementary material for: Antibodies-Abzymes with Antioxidant Activities in Two Th and 2D2 Experimental Autoimmune Encephalomyelitis Mice during the Development of EAE Pathology
Source: Molecules. 2022 Nov 3;27(21):7527. doi: 10.3390/molecules27217527 (PMC9656754; doi:10.3390/molecules27217527)
Supplement: Supplementary file 1 [file molecules-27-07527-s001.zip › molecules-1999026-supplementary.pdf]

molecules

MDPI

Supplementary methods

Article

Antibodies-Abzymes with Antioxidant Activities in Two Th and 2D2 Experimental Autoimmune Encephalomyelitis Mice during the Development of EAE Pathology

Anna S. Tolmacheva, Kseniya S. Aulova, Andrey E. Urusov, Vasilii B. Doronin and Georgy A. Nevinsky *

Institute of Chemical Biology and Fundamental Medicine, Siberian Division of the Russian Academy of Sciences, 630090 Novosibirsk, Russia
* Correspondence: nevinsky@niboch.nsc.ru

1. Immunization of mice (Part I)

The immunization of the C57BL/6, Th, and 2D2 mice with MOG was carried out as described in previously published protocols [1-3]. On day 1 (zero time), the mice were immunized by the injection of 10 µg of MOG per mouse in the back, two times in the left and right side using 20 µl of Freund's complete adjuvant containing Pertussis toxin (400 ng/mouse; *Mycobacterium tuberculosis*). The next day, an additional 20 µl of Pertussis Toxin (400 ng / mouse) was injected in a similar way.

2. IgG purification (Part II)

Electrophoretically and immunologically, the homogeneous mouse IgGs were obtained by the sequential chromatography of the serum proteins on Protein G-Sepharose and following the fast protein liquid chromatography (FPLC) gel filtration as described previously [1-3]. The serum protein (0.4–0.6 ml) was loaded onto a 1-ml protein G-Sepharose column which was equilibrated in buffer A (150 mM NaCl, 50 mM Tris-HCl, pH 7.5). The column was washed with buffer A to a zero optical density (A_{280}). The proteins adsorbed non-specifically were eluted with the same buffer (15 ml), but it contained 1% Triton X-100 and 0.3M NaCl, and the column was washed with buffer A to a zero optical density. The total IgGs fraction was eluted with 0.1 M glycine-HCl (pH 2.6), the column fractions were collected in cooled tubes containing 50 ml of 0.5M Tris-HCl (pH 9.0), and finally, each fraction was additionally neutralized with this buffer, which was concentrated for additional purification.

The purified IgG was incubated in acidic glycine-HCl buffer (pH 2.6) to disrupt the non-covalent interactions and subjected to FPLC gel filtration on a Superdex 200 HR 10/30 column (Pfizer, New York, NY) using the BioCA workstation (Applied Biosystems, Foster City, CA) [1-3]. The Abs were incubated for 20 min at 25 °C in 0.1 M buffer (pH 2.6) containing 0.3 M NaCl and then, subjected to the gel filtration on the column equilibrated in buffer A. The fractions of the separated IgGs were collected and dialyzed against 20mM Tris-HCl (pH 7.5) containing 50 mM NaCl.

In order to protect the Abs preparations from bacterial and viral contamination, they were filtered through Millex syringe-driven filter units (0.2 µm) and kept in sterilized tubes. The incubation of the standard bacterial medium which was performed by storing the Abs preparations did not lead to the formation of colonies.

2.1. Analysis of bone marrow progenitor cells in culture

The bone marrow samples were flushed out from the mouse femurs, and the colony-forming ability of the bone marrow cells was estimated as in [19-22]. Four dishes per

mouse (each containing 2×10^4 cells) were cultured in a standard methylcellulose-based M3434 medium that was specific for mouse cells (StemCell Technologies, Canada). The medium contained stem cell factor, interleukin (IL)-3, IL-6, and erythropoietin (EPO). Relative number of CFU-GM, CFU-E, BFU-E, and CFU-GEMM colonies were calculated after 14 days of sample incubation at 37°C and 5% CO_2 in a humidified incubator as described previously [17-21].

2.2. Analysis of lymphocyte proliferation

An analysis of the lymphocyte proliferation in vitro (sum of B and T cells) was carried out as in [19-22]. The cells (10^6/ml) isolated from the spleen, bone marrow, lymph nodes, and thymus were cultivated in 96-well flat-bottom plates (Trasadingen, Switzerland) containing RPMI-1640 medium supplemented with 10 mM HEPES buffer, 10% of fetal calf serum, 2 mM L-glutamine, 0.5 mM 2-mercaptoethanol, 100 µg/ml benzylpenicillin, and 80 µg/ml gentamicin. After a 64 hour incubation period, a solution (15 µl) containing 5 mg/ml MTT (tetrazolium dye MTT is 3-(4,5-dimethylthiazol-2-yl)-2,5-diphenyltetrazolium bromide) was added to each well, and the plates were incubated at 37°C for an additional 4 h. Then, the plates were centrifuged for 10 min at 1200×g, and the solutions were removed. The cells were precipitated by the addition of DMSO (200 µl); the mixtures were resuspended and incubated at 23 °C for 15 min in darkness. The analysis of the relative cell amount was performed spectrophotometrically at 492 nm.

3. Supplementary Figures

An analysis of the differentiation profiles of the bone marrow stem cells (BFU-E, CFU-E, CFU-GM, and CFU-GEMM) during the spontaneous and MOG-induced development of EAE in the C57BL/6, Th, and 2D2 mice was previously carried out. It was shown that in all of the cases, the immunization of the mice with MOG led to an acceleration of the development of EAE. However, the changes in the differentiation profiles of the stem cells in these mice during the development of EAE were significantly different. Below, the changes in the profiles of the stem cell differentiation in three types of mice (C57BL/6, Th, and 2D2) are given [1-4].

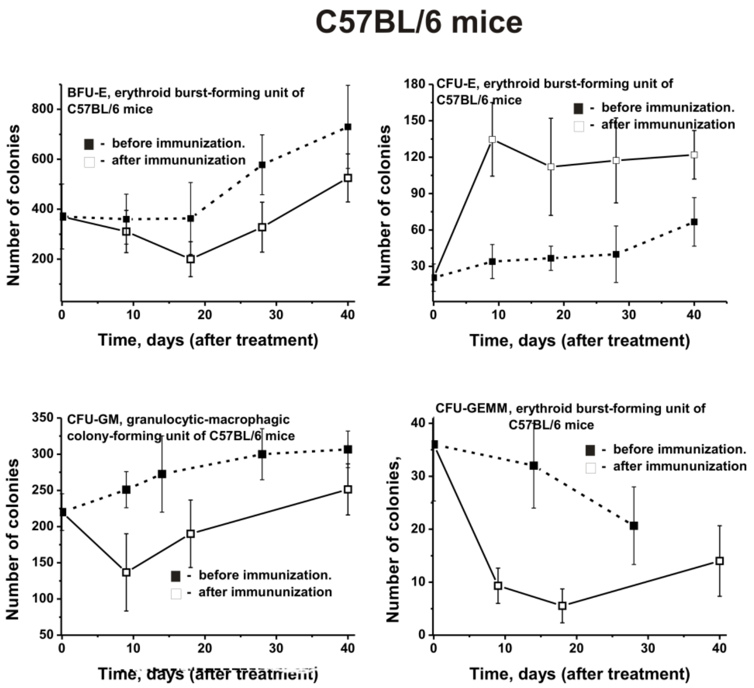

Figure S1. Changes over time of the average content of bone marrow progenitor colony-forming units for untreated and MOG-treated C57BL/6 mice (7 mice of each group): BFU-E, CFU-E, CFU-GM, and CFU-GEMM [1].

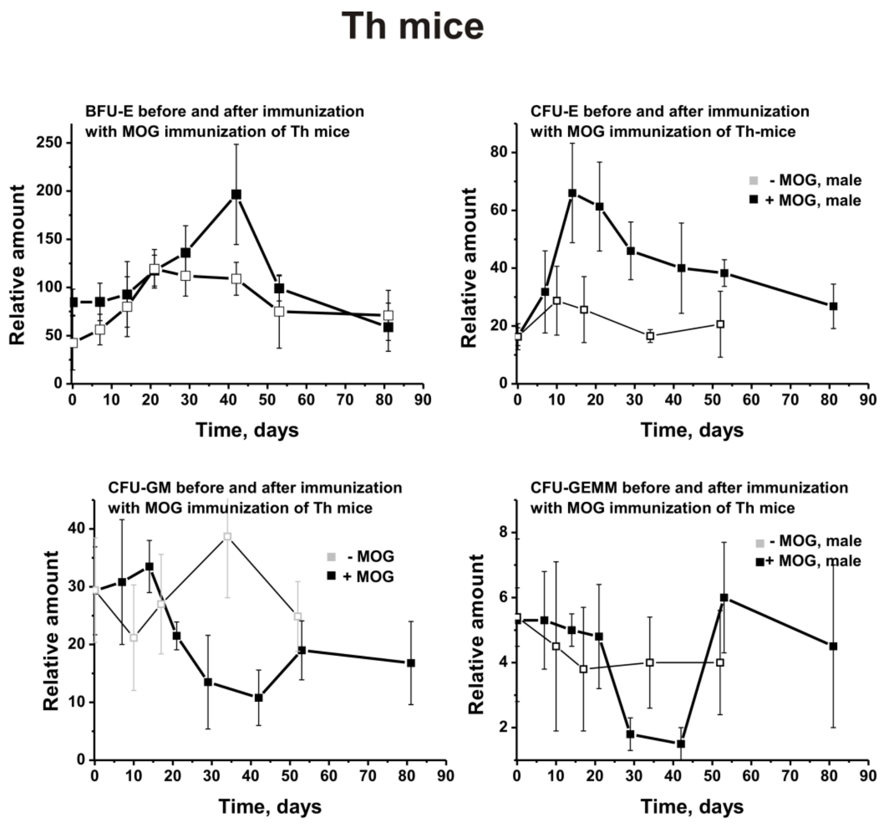

Figure S2. Changes over time of the average content of bone marrow progenitor colony-forming units (BFU-E, CFU-E, CFU-GM, and CFU-GEMM) for untreated and MOG-treated Th mice (7 mice of each group) [2,3].

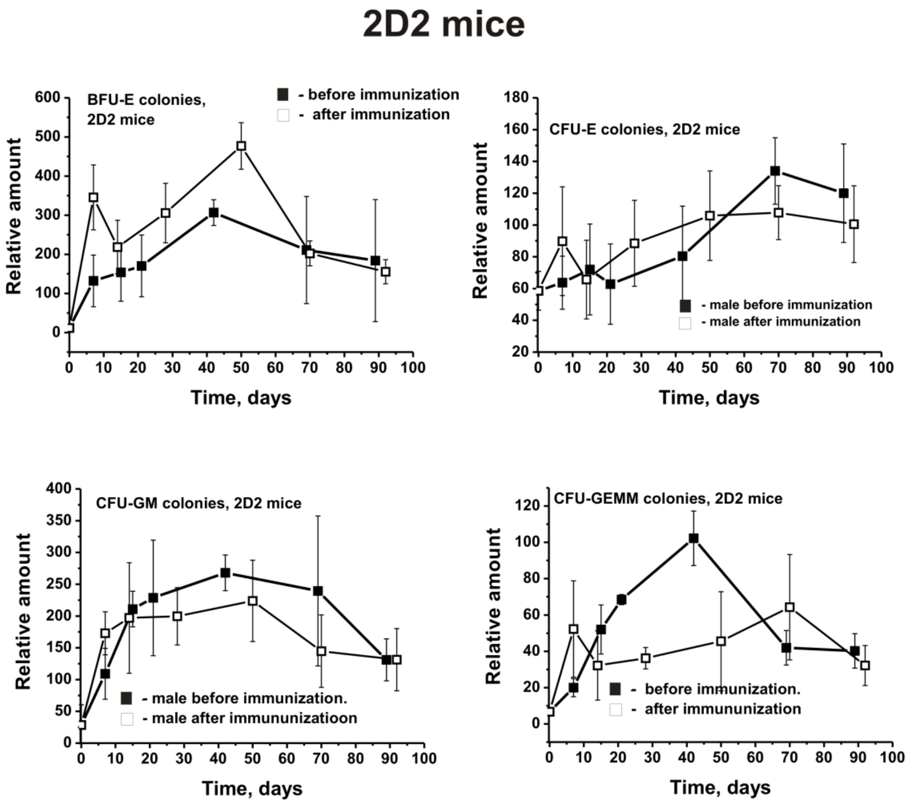

Figure S3. Changes over time of the average content of bone marrow progenitor colony-forming units (BFU-E, CFU-E, CFU-GM, and CFU-GEMM) for untreated and MOG-treated 2D2 mice (7 mice of each group) [4].

An analysis over time of the relative activity of the IgGs during the spontaneous and MOG-induced development of EAE in the C57BL/6, Th, and 2D2 mice was previously carried out [1-4]. It was shown that in all of the cases, the immunization of the mice with MOG led to a change in the relative activity of antibodies in the hydrolysis of DNA, MBP, and MOG. The figures present the changes in the activities in three types of mice (C57BL/6, Th, and 2D2), and these are given below [1-4].

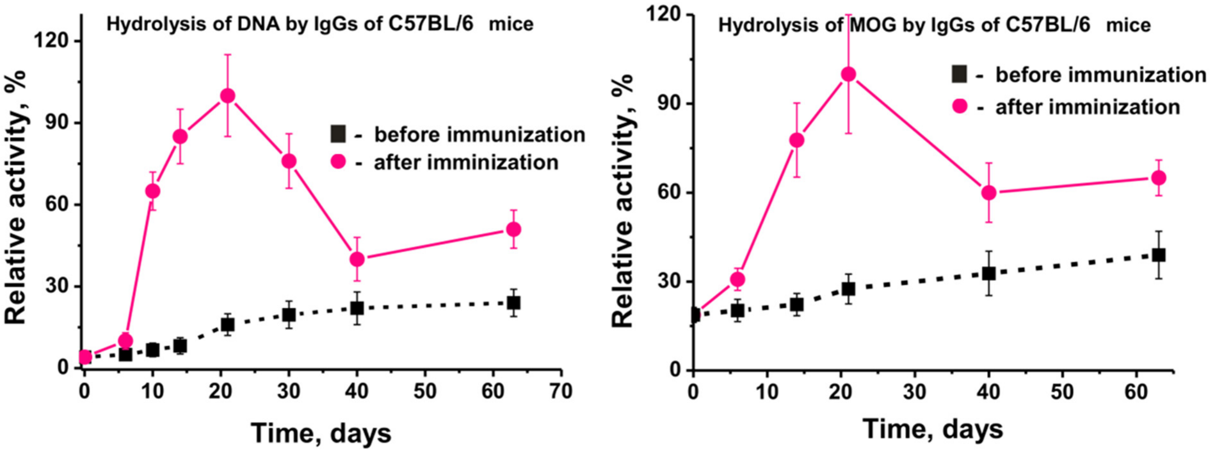

Figure S4. Changes over time of the average relative activities of IgGs of untreated and MOG-treated C57BL/6 mice (7 mice of each group) [1,2].

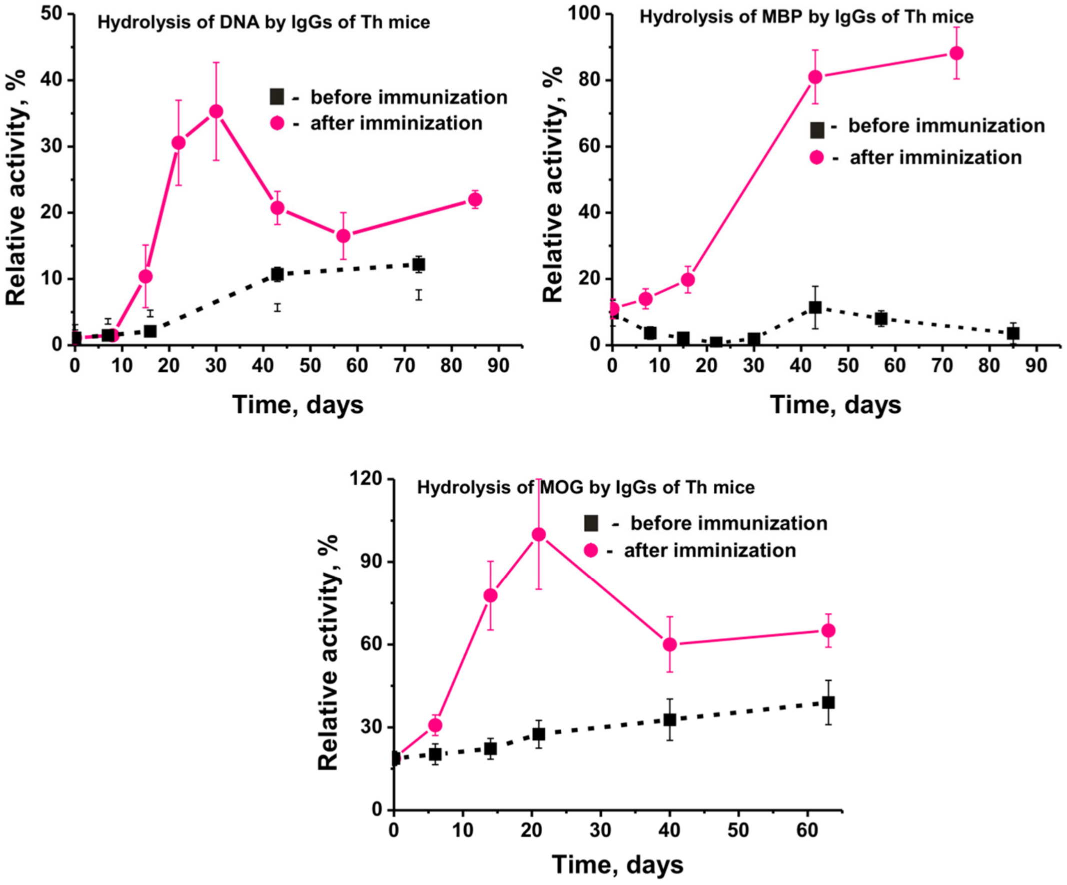

Figure S5. Changes over time of the average relative activities of IgGs of untreated and MOG-treated Th mice (7 mice of each group) [3].

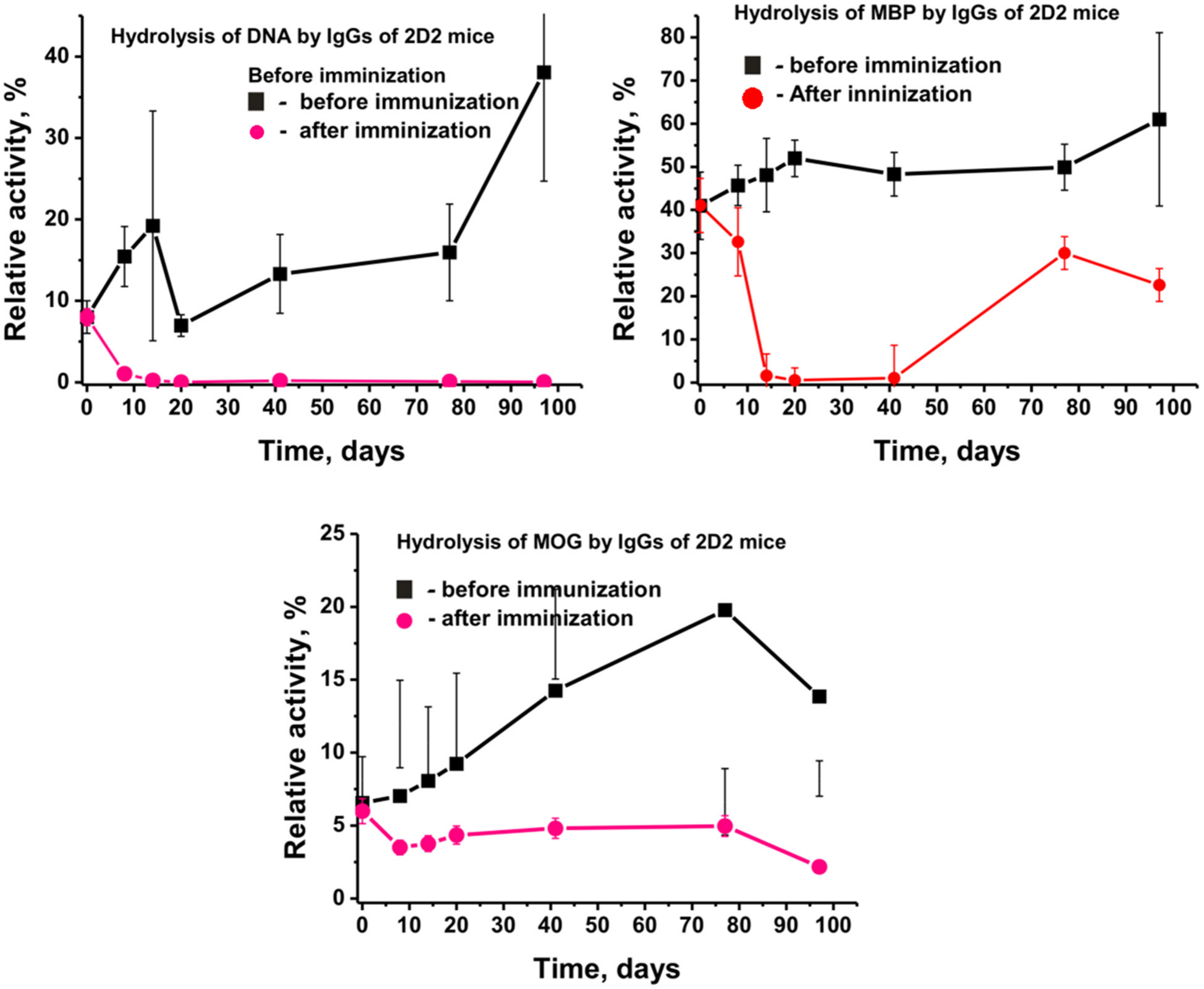

Figure S6. Changes over time of the average relative activities of IgGs of untreated and MOG-treated 2D2mice (7 mice of each group) [4].

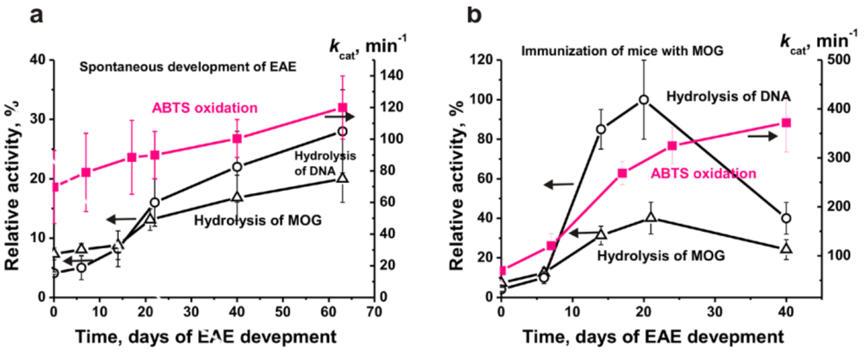

Figure S7. Changes over time of the average relative activities of IgGs of untreated (a) and MOG-treated (b) C57BL/6 mice (7 mice of each group) in the hydrolysis of DNA, MOG, and oxidation of ABTS [48].

References

1. Doronin, V.B.; Parkhomenko, T.A., Korablev, A., Toporkova, L.B., Lopatnikova, J.A., Alshevskaja, A.A.; Sennikov, S.V.; Buneva, V.N.; Budde, T.; Meuth, S.G.; et al. Changes in different parameters, lymphocyte proliferation and hematopoietic progenitor colony formation in EAE mice treated with myelin oligodendrocyte glycoprotein. *J. Cell Mol. Med.* **2016**, *20*, 81–94.

2. Aulova, K.S.; Urusov, A.E.; Toporkova, L.B.; Sedykh, S.E.; Shevchenko, Y.A., Tereshchenko, V.P.; Sennikov, S.V.; Budde, T.; Meuth, S.G.; Popova, N.A.; Orlovskaya, I.A.; Nevinsky, G.A. Production of Abzymes in Th, CBA, and C57BL/6 Mice before and after MOG Treatment: Comparing Changes in Cell Differentiation and Proliferation. *Biomolecules* **2019**,*10*, 53
3. Aulova, K.S.; Urusov, A.E.; Toporkova, L.B.; Sedykh, S.E.; Shevchenko, Y.A., Tereshchenko, V.P.; Sennikov, S.V.; Budde, T.; Meuth, S.G.; Orlovskaya, I.A.; Nevinsky, G.A. Catalytic antibodies in the bone marrow and other organs of Th mice during spontaneous development of experimental autoimmune encephalomyelitis associated with cell differentiation. *Mol. Biol. Rep.* **2021**, *48*, 1055-1068.
1. Aulova, K.S.; Urusov, A.E. Toporkova, L.B.; Sedykh, S.E.; Shevchenko, J.A.; Tereshchenko, V.P.; Sennikov, S.V.; Orlovskaya, I.A.; Nevinsky, G.A. Cell Differentiation and Proliferation in the Bone Marrow and Other Organs of 2D2 Mice during Spontaneous Development of EAE Leading to the Production of Abzymes. *Molecules*. **2022**, *27*, 2195.
